# Matched Field Processing Based on Bayesian Estimation

**DOI:** 10.3390/s20051374

**Published:** 2020-03-02

**Authors:** Guolei Zhu, Yingmin Wang, Qi Wang

**Affiliations:** School of Marine Science and Technology, Northwestern Polytechnical University, Xi’an 710072, China; flyingscott@nwpu.edu.cn (G.Z.); ywang@nwpu.edu.cn (Y.W.)

**Keywords:** underwater signal processing, AMFP, posterior probability density, robustness

## Abstract

In order to improve the robustness and positioning accuracy of the matched field processing (MFP) in underwater acoustic systems, we propose a conditional probability constraint matched field processing (MFP-CPC) algorithm in this paper, which protects the main-lobe and suppresses the side-lobe to the AMFP by the constraint parameters, such as the posterior probability density of source locations obtained by Bayesian criterion under the assumption of white Gaussian noise. Under such constraint, the proposed MFP-CPC algorithm not only has the same merit of a high resolution as AMFP but also improves the robustness. To evaluate the algorithm, the simulated and experimental data in an uncertain shallow ocean environment is used. From the results, MFP-CPC is robust to the moored source, as well as the moving source. In addition, the localization and tracking performances of using the proposed algorithm are consistent with the trajectory of the moving source.

## 1. Introduction

Targets detection and recognition are popular research topics in both civilian and military applications. Fuzzy linguistic modelling is recently proposed for information extraction [1]. However, it is difficult to employ fuzzy linguistic modelling in underwater target detection due to the computational burden. Matched field processing (MFP) technology successfully combines the physical characteristics of underwater acoustic channels and the traditional signal processing algorithm, thus it is widely used in the underwater target passive location and acoustic parameters inversion, etc. [2]. In MFP, many techniques are potentially used for signal processing, parameters estimation, and signal detection, such as neural network-based feature extraction, knowledge-based Bayesian filtering, and hidden Markov model [3,4,5]. In most cases, the sound propagation model (such as the normal mode model, the parabolic equation, and the ray, etc.) is used to build a replica vector of the field in the observation sea area [6], and then match the measured field with the replica to estimate the location of the source or the channel information [7,8]. MFP is mainly divided into two categories: One is the conventional matching field processor (conventional MFP, CMFP [9]), also known as the Bartlett processor; another is adaptive matched field processor (adaptive MFP, AMFP), the representative is the minimum variance distortionless response (MVDR) processor [10,11]. Bartlett is robustness, but it is difficult to separate the main-lobe from side-lobe for its side-lobe is much higher. MVDR provides maximum array gain for its excellent side-lobe suppression characteristics, but its performance is not robust when the underwater acoustic channel model is different from the actual conditions, that is to say, there is a mismatch.

Generally, the parameters of the environment vary over the time. In order to reduce the impact of the parameter changes, some researchers try to improve the robustness of the algorithm [12,13,14]. Researchers in [15,16,17] have proposed many tolerance AMFP method based on the MVDR, such as the minimum variance-neighborhood location constraints (MV-NLC), the minimum variance-environmental perturbation constraints (MV-EPC), and the sector focusing (SF), etc.

MV-NLC protects the main lobe and overcomes the environment mismatch with small-scale position constraints, but the effectiveness of the algorithm relies on a similar degree of the environment mismatch and the position change of the sound source. In waveguide with the same sound velocity, the deep sea error corresponding to the sound source location changes. However, other types of environmental mismatch, such as sound velocity profile and geoacoustic parameters mismatch is not similar, in this case, the MV-NLC will fail; MV-EPC protects the main-lobe by using the first and second-order statistical properties of the replica of signal correlation matrix within the scope of environmental parameter perturbation, with heavy computation. SF constructed a projection matrix by multiple replicas in some areas, using the projection matrix to eliminate the influence on the MFP made by noise and environment mismatch, but it is difficult to choose the sector size. In addition, there are some other robust AMFP algorithms, such as reduced-order MV-EPC, environment perturbation constraints SF, etc.

Our algorithm uses the statistical methods to reduce the impact of environmental disturbance, which is powerful in target detection [5,18]. We use conditional probability constraint to provide a certain degree of main-lobe protection and side-lobe suppression to the AMFP, and obtained good tolerance in mismatch environment. We verify the performance of our algorithm by the simulation data of a typical mismatch environment general mismatch model (GENLMIS), which is published in the Naval Research Laboratory seminar in 1993 [19]. Finally, through the processing and analysis with the ocean experimental data from the SACLANT research center in 1993, making a comparison of the Bartlett, MVDR, and MFP-CPC location capability, verifying the validity and robustness of the MFP-CPC algorithm in the uncertain environment.

The notations in this paper are summarized and shown in Table 1, and the rest of the paper is organized as follows. Section 2 describes how the MFP-CPC algorithm is derived. Section 3 introduces the simulation, and the simulation results are analyzed. The simulation results are verified in Section 4 by the real ocean experimental data. Finally, conclusions are drawn in Section 5. In this work, we represent vectors with lower-case letters, and boldfaced upper-case letters are reserved for matrices.

## 2. Robust MFP in the Uncertain Environment

### 2.1. Data Model

Assuming ω is the frequency of the sound source, m is the position parameter (include distance r and depth z), and the sound propagation channel parameter set is ψ, then the sound pressure vector that is received by vertical linear array of N sensors in the frequency domain can be expressed as:

(1) x(ω)=a(ω)s(ω,m,ψ)+n(ω)

where n(ω) is the noise vector, assume it is zero-mean additive white Gaussian noise, a(ω) is the amplitude of the complex signal, s(ω,m,ψ) is the channel transmission function, s(ω,m,ψ) can be expressed as below by the Normal mode theory [20,21]:

(2) $$s\left(\omega ,m,\psi \right)=-\frac{i}{\rho (z_{s})\sqrt{8\pi r}}e^{-i\pi /4}\sum_{l=1}^{L}u_{l}(z_{s})u_{l}(z)\frac{e^{ik_{rl}r}}{\sqrt{k_{rl}}}$$

Here, $z_{s}$ is the target source depth, $k_{rl}$ is wave number, $u_{l}$ is normal mode function.

### 2.2. MVDR

Generally, the output of the MFP B(m) is composed of sampling covariance matrix R and weight vector w.

(3) $$B(m)=w{\left(m\right)}^{H}Rw(m)$$

The sampling covariance matrix R can be expressed by maximum likelihood estimation of K frequency snapshots such as data model of Equation (1):

(4) $$R=\frac{1}{K}\sum_{k=1}^{K}x_{k}x_{k}^{H}$$

In order to suppress the side-lobe and improve the resolution, an AMFP method is introduced in the paper [10,22,23], known as the minimum variance distortionless response (MVDR) processor, its mathematical expression is:

(5) $$\underset{w}{\min}w^{H}Rw,\;s.t.\;w^{H}w_{c}=1$$

Here, the $w_{c}=s\left(\omega ,m_{c},\psi \right)$ is N×1 dimension replica vector at the observation point $m_{c}$. As can be seen from the mathematical expression of the algorithm, it constructs a unity gain weight vector of the observation direction to a minimum and the weighted array output in the other direction, so as to inhibit the side-lobe and improve the resolution.

Using the Lagrange multiplier method in solving this optimization problem, getting the weight vector and the power output of the MVDR are shown as below:

(6) $$w_{\mathrm{MVDR}}=\frac{R^{-1}w_{c}}{w_{c}^{H}R^{-1}w_{c}}$$

(7) $$B_{\mathrm{MVDR}}=\frac{1}{w_{c}^{H}R^{-1}w_{c}}$$

The MVDR not only has better array gain but also has better main-lobe and side-lobe than the Bartlett, but when underwater acoustic environment perturbation, especially under the condition of high SNR, the AMFP is easy to have a serious “target suppression” phenomenon, so it is much less robust than the Bartlett.

### 2.3. MFP-CPC

According to the Bayesian criterion, the posterior probability density function (PDF) of the sound source location parameters can be expressed as [24,25,26]:

(8) $$p(m|x)=\frac{p(x|m)p(m)}{p(x)}$$

where p(m) and p(x) are respectively, the PDF of the sound source location m and the PDF of the measurement field x. When the measurement field x is known, conditional probability density p(x|m) is the function of the location parameter m.

For the data model shown in Equation (1), because the noise of every element of the N sensors vertical linear array is independent identically as distributed additive white Gaussian noise, so we can get the conditional probability density p(x|m), that is the likelihood function can be expressed as below:

(9) $$p(x|m)=\frac{1}{{\left(\sqrt{2\pi }\sigma_{n}\right)}^{N}}\exp\left[-\frac{{\left|x-as\left(\omega ,m,\psi \right)\right|}^{2}}{2\sigma_{n}^{2}}\right]$$

where $\sigma_{n}^{2}$ is the power of noise.

Let ∂p(x|m)/∂a=0, the maximum likelihood estimation of a is shown as below:

(10) $$\overset{⌢}{a}=\frac{x^{H}s\left(\omega ,m,\psi \right)}{{\left|s\left(\omega ,m,\psi \right)\right|}^{2}}$$

Substituting Equation (10) into Equation (9) and putting s(ω,m,ψ) shorthand for s, yields the likelihood function:

(11) $$p(x|m)=\frac{1}{{\left(\sqrt{2\pi }\sigma_{n}\right)}^{N}}\exp\left[\frac{s^{H}Rs-{\left|x\right|}^{2}\cdot {\left|s\right|}^{2}}{2\sigma_{n}^{2}\cdot {\left|s\right|}^{2}}\right]$$

Substituting Equation (11) into Equation (8), we can get the posterior PDF by Bayesian criterion shown as follows:

(12) $$p(m|x)=\frac{1}{{\left(\sqrt{2\pi }\sigma_{n}\right)}^{N}}\exp\left[\frac{s^{H}Rs-{\left|x\right|}^{2}\cdot {\left|s\right|}^{2}}{2\sigma_{n}^{2}\cdot {\left|s\right|}^{2}}\right]\cdot \frac{p(m)}{p(x)}$$

Assume that in the observation sea area, the target can appear at any position randomly, so the probability of the target that appears in this area is uniformly distributed, so the p(m) is a constant. When the prior PDF p(x) is unknown, in order to ensure the integration of the posterior PDF equal to 1, that is:

(13) $$\sum_{m}p\left(m|x\right)=1$$

We defined a normalization constant $C_{x}$, then get the constant $C_{x}$ as shown below:

(14) $$C_{x}=\frac{1}{\sum_{m}\exp\left[\frac{s^{H}Rs-{\left|x\right|}^{2}\cdot {\left|s\right|}^{2}}{2\sigma_{n}^{2}\cdot {\left|s\right|}^{2}}\right]}$$

Therefore, after we got the posterior PDF of the location parameters, we can construct the MFP as Equation (15), named as MFP with the conditional probability constraint (MFP-CPC):

(15) $$B_{\mathrm{CPC}}=p(m|x)\cdot B_{A}=C_{x}\exp\left[\frac{s^{H}Rs-{\left|x\right|}^{2}\cdot {\left|s\right|}^{2}}{2\sigma_{n}^{2}\cdot {\left|s\right|}^{2}}\right]\cdot B_{A}$$

where $B_{A}$ is the power output of AMFP. From the output expression of MFP-CPC, it is clear that the MFP-CPC using prior knowledge about the environment and introducing the Bayesian criteria to the algorithm, is a kind of combining method by data-driven and the base model. Taking the AMFP as the basic unit, the algorithm has a general expression.

## 3. Simulation and Analysis

**Figure 1:**
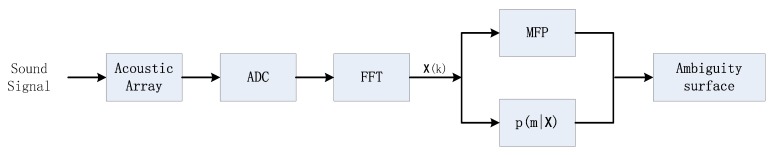
Block diagram of MFP-CPC.

In this section, the simulation results are shown and discussed. The detailed steps for setting up the simulation is illustrated in Figure 1. To set up the simulation, firstly we obtain the posterior PDF p(m|x) as shown in Equation (12), secondly we derive the classic MVDR parameter, $B_{MVDR}$, as shown in Equation (7), finally we use p(m|x) to constraint the $B_{MVDR}$ as shown in Equation (15), then we get the result of $B_{cpc}$.

### 3.1. Simulation Model

In May 1993, the seminar of Naval Research Laboratory presented several typical shallow sea environment model and the simulation data for the researchers to use [19], its purpose is to be able to compare the performance of the different environment matched field inversion and positioning of the algorithm objectively and fair. The Kraken normal mode model is used to calculate the replica vector. The MATLAB program of Kraken is given by Naval Research Laboratory [27], substituting environmental parameters into Equation (2), then we can get the channel transmission function. GENLMIS is used to verify the performance of the algorithm in the presence of colored noise environment and with parameter mismatch, it is a simulation case of severe mismatch. In this paper, the simulation and analysis are based on the data.

A vertical linear array with 20 sensors is used to receive a signal in the simulation, the sound source frequency is 250 Hz. The depth of the first sensor is 5 m, the depth of the last sensor is 100 m, with 5 m equal spacing. The distance of the observation area from 5 to 10 km, distance step 20 m; depth from 1 to 100 m, depth step 1 m.

**Figure 2:**
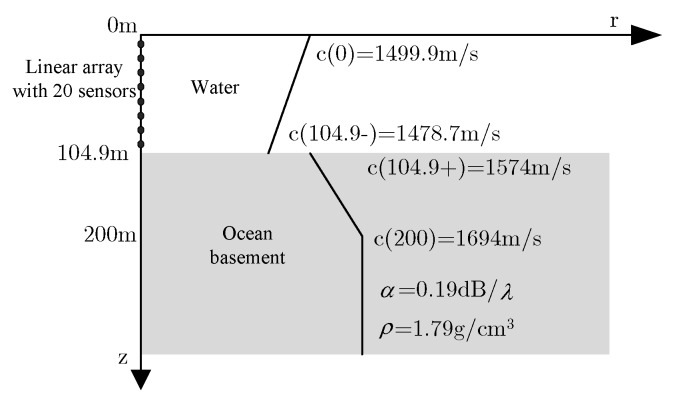
GENLMIS real environment model.

The real environment parameter model GENLMIS is shown in Figure 2, that uses this environment model to construct a measured field. There are three kinds of sound source under this environment: (1) Sound source is located at (6.2 km, 92 m), SNR is 40 dB; (2) sound source is located at (9.0 km, 74 m), SNR is 10 dB; (3) sound source is located at (7.2 km, 16 m), SNR is −5 dB.

### 3.2. Simulation Result without Environment Mismatch

**Figure 3:**
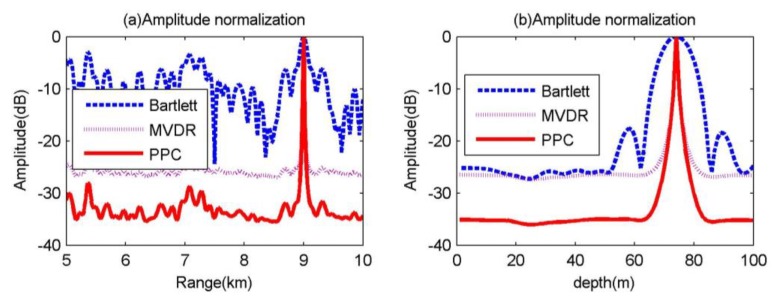
Slices of localization results for the three matched field processing (MFP) methods.

Assuming the sound source is located at (9.0 km, 74 m), the SNR is 10 dB, the accurate environment parameter model is shown in Figure 2. The Bartlett, MVDR, and MFP-CPC are used to do match field processing, respectively, the slices of the source is shown in Figure 3, these three kinds of MFP can pinpoint the target successfully according to the results in Figure 3, the MVDR and MFP-CPC have the more narrow main-lobe and lower side-lobe. When the number of sensors N is 20 and is exactly matching, the theory output power of the MFP is 10lg(N)=13dB, the output power of Bartlett and MVDR at (9.0 km, 74 m) are approximate to 13 dB, agreeing with the theoretical value. However, the output power of MFP-CPC is far less than the two before, the main reason is that the posterior probability has an influence on the output of MFP.

The comparison for the results after amplitude normalization of the three kinds of processor is shown in Figure 3a,b. In Figure 3, the main-lobe of MFP-CPC and MVDR are coinciding, the −3 dB main-lobe width for the distance are all 20 m, the −3 dB main-lobe width for the depth is all 1.5 m, but Bartlett’s main-lobe width for the distance and depth is 100 and 7 m, respectively. In Figure 3a, for the distance, the highest side-lobe of MVDR is about 35 dB less than the Bartlett and the highest side-lobe of MFP-CPC is about 6 dB less than MVDR. In Figure 3b, for the depth, the side-lobe of MVDR and MFP-CPC are not able to be distinguished, they are lost in the background, the background of MVDR is about 24 dB less than the highest side-lobe of Bartlett and the background of MFP-CPC is about 8 dB less than the background of MVDR.

From the simulation result without environment mismatch, it is clear that the localization performance of MFP-CPC is much better than the Bartlett, the main-lobe of MFP-CPC is the same as MVDR, and the side-lobe of MFP-CPC is about 6 dB less than MVDR, the background of MFP-CPC is about 8 dB less than MVDR.

### 3.3. Simulation Result with Environment Mismatch

**Figure 4:**
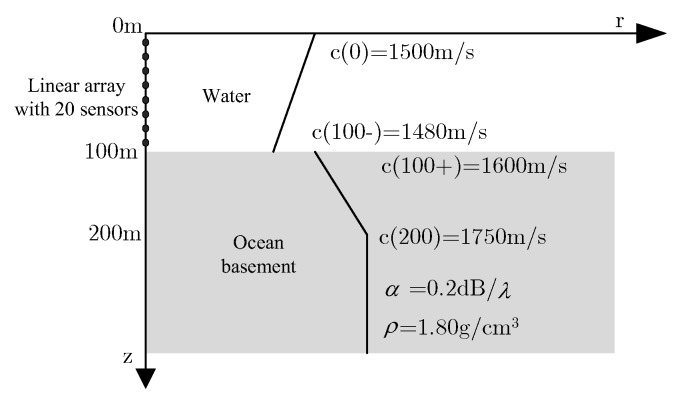
GENLMIS environment model with mismatch.

The measured field is still calculated by the environment parameter model as shown in Figure 2, assuming that the geoacoustic parameters are uncertain, so the real environment parameters are only able to get a priori knowledge about the environmental parameters as shown in Figure 4 according to the experience. Compared with the real environment model shown in Figure 2, the sound velocity of the surface of ocean mismatch −0.1 m/s, the sound velocity of the bottom of ocean mismatch −1.3 m/s, the sound velocity of the upper surface of ocean basement mismatch −26 m/s, the sound velocity of the middle of ocean basement mismatch −56 m/s, the sound velocity of the bottom of ocean basement mismatch −56 m/s, the attenuation coefficient of the ocean basement mismatch −0.01 dB/λ, the density of the ocean basement mismatch −0.01 g/cm^3^, depth of the sea mismatch 4.9 m, and there is colored noise in the measured field data of the GENLMIS model.

**Figure 5:**
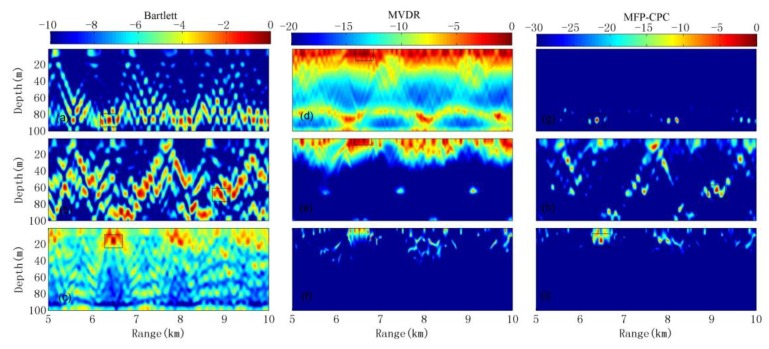
Localization results for the three MFP methods under GENLMIS environment model.

Under the GENLMIS model with environment parameters mismatch, the ambiguity surface of the location result by the three kinds of MFP for the three sound sources introduced in Section 3.1 is shown in Figure 5. Classified according to the type of MFP: Figure 5a–c is the location results of Bartlett; Figure 5d–f shows the location results of MVDR; Figure 5g–i is the location results of MFP-CPC. Classified according to the sound source: Figure 5a–g is the location results of first sound source with 40 dB SNR; Figure 5b,e,h is the location results of second sound source with 10 dB SNR; Figure 5c,f,i is the location results of third sound source with −5 dB SNR. The main-lobe value in the small rectangular box in each sub-image is the estimation of sound source location by the three kinds of MFP in Figure 5.

When the SNR is 40 and 10 dB, the Bartlett and MFP-CPC give the correct location results, but in the ambiguity surface of Bartlett, there are many side-lobes amplitude that are close to the main-lobes. In Figure 5a, the main-lobe at (6.2 km, 92 m) is bigger than the maximum side-lobe at (8.2 km, 92 m) less than 2 dB, in Figure 5b the main-lobe at (9.1 km, 72 m) is bigger than the maximum side-lobe at (7.2 km, 70 m) less than 1 dB, it is very hard to distinguish. However, in Figure 5g, the main-lobe of MFP-CPC is about 6 dB bigger than the maximum side-lobe. In Figure 5h, the maximum side-lobe is located at (7.7 km, 16 m), it is not same as the position of the maximum side-lobe in Figure 5b, as you can see it is because of the influence of MVDR from Figure 5e, and in Figure 5h, the main-lobe at (9.1 km, 71 m) is still about 3 dB bigger than the maximum side-lobe, it is obvious that the MFP-CPC’s side-lobe compression performance is better. MVDR has severe “suppression” phenomenon under the condition of the two kinds of SNR, there are many peaks that appear at the surface of the sea, it means the MVDR is most sensitive to environmental mismatch, but the good thing is the peak can still be seen at the real sound source location. When the SNR is -5 dB, all the three kinds of MFP failure at this time, the influence of colored noise and environmental mismatch is most obviously, the positioning of the false target that is blurred by MVDR. Although Bartlett and MFP-CPC only have false targets at (6.5 km, 16 m), the false targets are clear. The contrast of the location results of Bartlett and MFP-CPC can be seen even in serious mismatch cases, their ambiguity of the location results near the real target also have a certain similarity, that is they have similar robustness.

## 4. Verification by Ocean Experimental Data

### 4.1. Experiment Parameter

**Figure 6:**
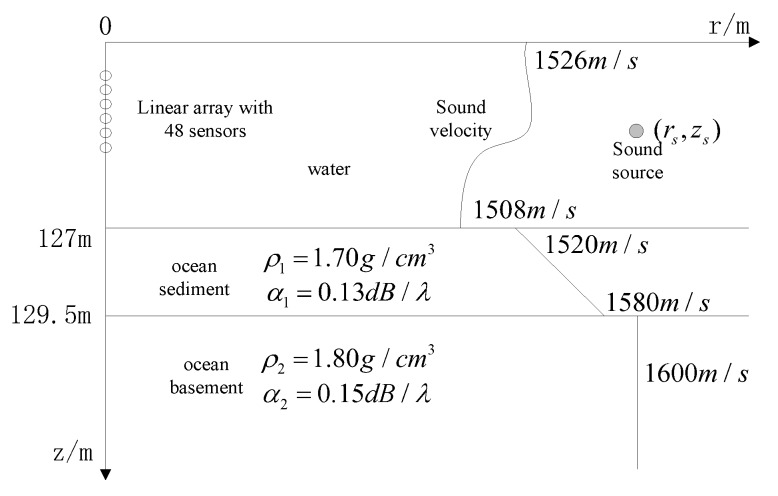
Environmental model in shallow water.

The ocean near the north island of Elba of the west coast of Italy is a typical shallow water environment, on 26 and 27 October, 1993, the SACLANT research center was doing an ocean experiment for two days at the ocean [28]. In this paper, we used the experimental data to do processing and analysis. The array and environment parameters for the experiment are described in Figure 6. The bathymetry (125.5–128.5 m); upper sediment sound-speed (1450–1550 m/s); lower sediment sound-speed (1500–1600 m/s); sediment density (1.2–2.2 g/cm^3^); sediment attenuation (0.0–0.4 dB/λ); sediment thickness (0.0-0.6 m); sub-bottom sound-speed (1550–1650 m/s); sub-bottom density (1.2–2.2 g/cm^3^); sub-bottom attenuation (0.0–0.4 dB/λ).

During the experiment on 26 October, the sound source moored in 5500 ± 200 m distance from the receiving array, the depth is about 79 m. On 27 October, the sound source was towed by a tugboat with 3 km speed, navigation in a straight line. The depth is about 65 m, the initial distance from the receiving array is about 5.9 km. The experiment collects the signals received from the linear array for 10 min. During the two days experiment, the acoustic signal model is slightly different, on 26 October, the signal is a continuous signal, on 27 October, the signals were not continuous, they were on for 30 out of every 60 s, we used the experimental data to verify the performance of the MFP-CPC.

According to Equation (4), we used the data of the first 25 s to make 25 snapshots, the average of these snapshots is the maximum likelihood estimation of the sampling covariance matrix. The length of the snapshot is 2 s, the overlap between the two snapshots is 50%, for a 1 kHz sampling rate, each snapshot contains 2000 sampling points.

### 4.2. Result of Experimental Data

**Figure 7:**
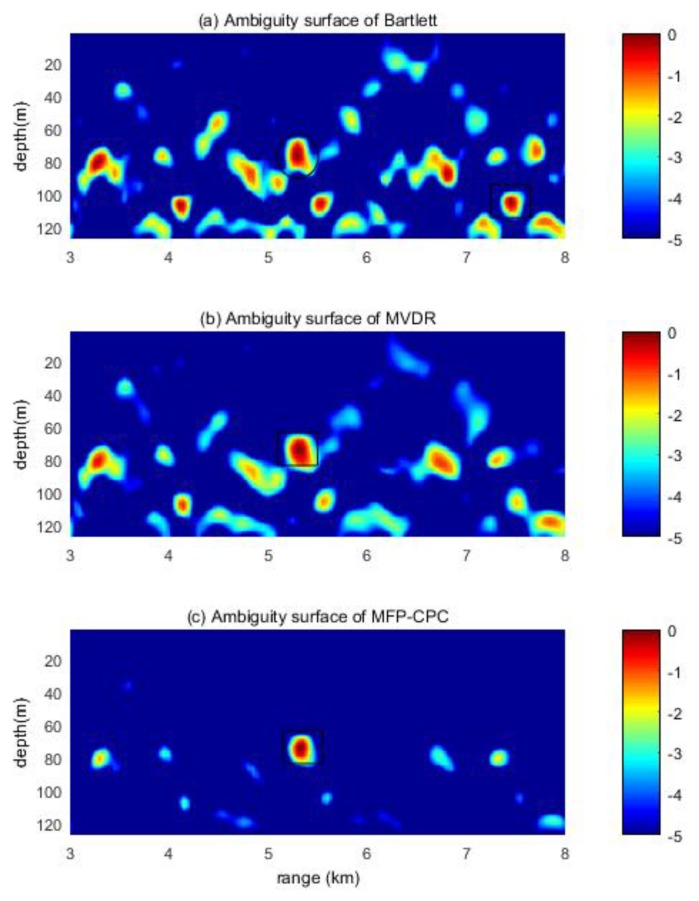
Ambiguity surfaces of the moored source at the first minute.

The location result of the moored source at the first minute is shown in Figure 7. The rectangle area is the position of the peak amplitude on the ambiguity surface, it is also the location result of the MFP, the ellipse area is the position of the real target, we only draw a rectangle when the ellipse and rectangle overlapped. If the peak amplitude position of the ambiguity surface is near the real target (depth around ±10 m, the distance around ±500 m), that suggests the localization success.

It is clear that the location result of Bartlett is wrong in Figure 7a, the error result is (104.7 m, 7450 m), there also is a peak at the real position. The location result (73.5 m, 5300 m) of MVDR is shown in Figure 7b, it is correct, but the amplitude of the side-lobe is a little bit higher, only around 1 dB lower than the main-lobe. Figure 7c is the ambiguity surface of MFP-CPC, we can clearly find the target and there is no confusion between the target and side-lobe.

**Figure 8:**
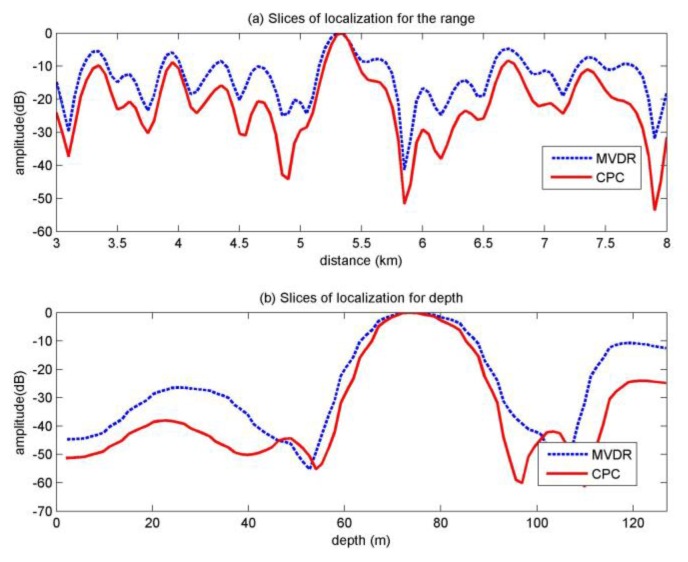
Slices of the moored source at the first minute.

The slices of the moored source at the first minute are shown in Figure 8, under the real uncertain environment, the Bartlett is failed to localization, thus we only compare the results of MVDR and MFP-CPC. From Figure 8, it is clear that the MFP-CPC has the more narrow main-lobe and lower side-lobe. The location results of 2 to 10 min are similar to the result of the first minute, the main difference is that Bartlett gives the correct results at 2, 5, 7, 8 min, the location results are all around (76.1 m, 5300 m). Both MVDR and MFP-CPC give the correct results during the 10 min, the results are the same as in the first minute.

In order to make a quantitative evaluation for the three kinds of MFP, we introduce two parameters for performance evaluation, one is signal to interference noise ratio (SINR), the other is the peak to background ratio (PBR). The SINR quantizes the differentiation between the main-lobe and the side-lobe, and it will be easier to separate the main-lobe from the side-lobe for a larger SINR; the PBR quantizes the differentiation between the main-lobe and background, it will be easier to find out the main-lobe when the PBR is bigger.

We quantitively make the comparison for the location results of the three kinds of MFP at the second minute in Table 2. For the depth localization, the result of Bartlett is closer to the target depth, but the result difference to the other two processors are only a depth of a grid. For the distance localization, the results are the same for the three processors, but the SINR and PBR of the MVDR are about 1 dB higher than the Bartlett, and the SINR and PBR of the MFP-CPC are about 2.5 dB higher than the MVDR. From the numerical results of the moored source at the second minute, we can see that MFP-CPC has an optimal localization performance, then for the MVDR, the Bartlett is the worst.

**Figure 9:**
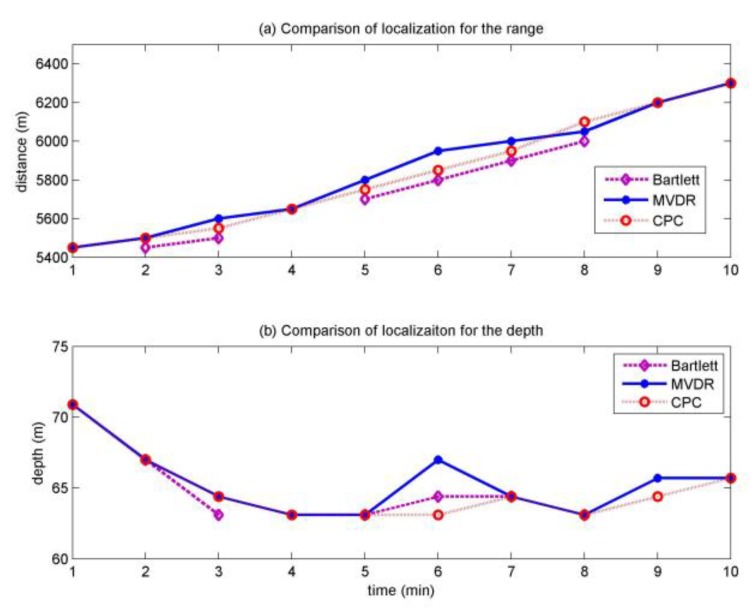
Tracking of the moving source in the whole 10 min.

The curve of localization results of the moving source in 10 min on 27 October is shown in Figure 9. During the 10 min, the Bartlett gives the correct results at 2, 3, 5, 6, 7, 8 min, the other time failed, the error localization results are not shown in Figure 9. The MVDR and MFP-CPC give the correct results in the whole 10 min, we can see that the motion curve of MFP-CPC is more like a uniform linear motion as shown in Figure 9, the slope of the curve of MFP-CPC is about 1.6 m/s, the MFP-CPC is the best consistent with the speed and trajectory of the moving source.

The distance localization result is around 500 m closer than the real target as shown in Figure 9a. It is perhaps that the water depth around the source is 13 m deep than 127 m, it is discussed in detail in article [28], the distance localization result of this article is equal to article [28]. In Figure 9b, the depth localization results of the three processors at the first minute are around 7 and 6 m error, at the sixth minute, the error of MVDR is about 3 m, the error of the other two processors is 1 m, at the other time, the depth localization error of the three processors are all within 2 m. Since the moving source is towed behind the ship, for the first and second minutes, at the beginning of the movement, the tension of the cable which connects the source is too small and unstable, thus causing the deep source. During this period, the depth localization results of the three processors are the same, so we can consider that the depth of the source is about 71 m. For the sixth and ninth minute, the tugboat moving smoothly, the depth localization difference of the three processors is mainly due to the ups and downs of sea waves; the uncertainty of the environment and array element position error. Under this condition, the localization performance heavily depends on the robustness of the algorithm. It is clear that the localization result of MFP-CPC is more accurate than MVDR, the Bartlett is failed at ninth minute.

**Figure 10:**
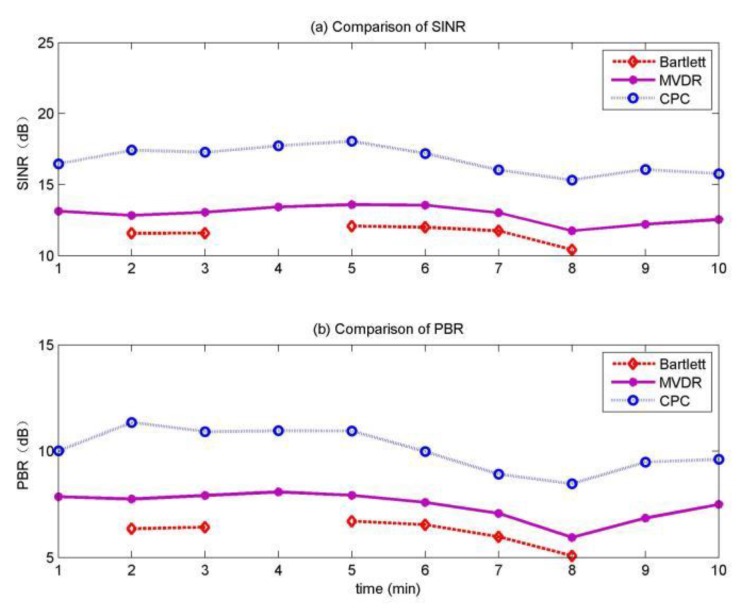
Localization performance for the moving source.

The SINR curve for the moving source in the 10 min is shown in Figure 10a when giving the correct localization results, the SINR of MVDR is about 1.5 dB higher than Bartlett, and the SINR of MFP-CPC is about 3 dB higher than MVDR. The PBR curve for the moving source in the 10 min is shown in Figure 10b, the PBR of MVDR is about 1.7 dB higher than Bartlett, the PBR of MFP-CPC is about 3 dB higher than MVDR. So not only it is easiest to distinguish main-lobe from side-lobe by the MFP-CPC, but also the main-lobe and background difference is the largest of MFP-CPC. It is clear that the MFP-PCPC has the best localization performance as shown in Figure 10.

## 5. Conclusions

The mismatch between the environment model and the real environment always leads to a serious performance penalty of MFP, especially in AMFP due to the uncertainty of the prior environment information. In order to solve this problem, a conditional probability constraint matched field processing (MFP-CPC) algorithm is proposed. For MFP-CPC, it derives the posterior probability density of the source locations by the field measurement data received by the vertical array, then uses the posterior probability density to protect the main-lobe of AMFP and suppresses the side-lobe. From the simulation result without environment mismatch, it is clear that the main-lobe width of MFP-CPC is the same as MVDR, and the side-lobe of MFP-CPC is about 6–8 dB less than MVDR. From the simulation result with the environment mismatch, it is clear that the MFP-CPC and Bartlett have a similar robustness.

For a typical uncertain shallow environment, the analysis results of ocean experimental data for the moving sound source show that MFP-CPC can restrain location errors caused by physical factors, such as, the wind and waves, the array element position, and the movement of the source, especially in the process of tracking for 10 min, the range and position curve accord well with the moving trail of the sound source.

In addition, the conditional probability constraints algorithm is relatively simple compared to other algorithms [12,13,14] to improve the robustness, and the expression is versatile and general. The adaptive unit of the algorithm can be replaced, so as to further improve its robustness. However, when calculating the posterior probability, the priori environmental information is required, therefore, the mismatch problems still exist. Compared with AMFP, the robustness of using the proposed algorithm is improved. 

In future work, to reduce the effects of the channel interferences on target detection, the method proposed in [29] is potentially applied and can be used to enhance the system performance.

## Figures and Tables

**Table 1:** Notation list.

Symbol	Description
ω	frequency of sound source
r	distance
z	depth
ψ	sound propagation channel parameter set
x(ω)	sound pressure vector
a(ω)	amplitude of the complex signal
n(ω)	noise vector
s(ω,m,ψ)	channel transmission function
k	wave number
$u_{l}\left(z\right)$	Normal mode function
B(m)	output of the MFP
R	sampling covariance matrix
ω(m)	weight vector
p(m)	the PDF of the sound source location
p(x)	the PDF of the measurement field
p(x|m)	conditional probability density
p(m|x)	posterior probability density function

**Table 2:** Results of the moored source at the second minute.

Processor	Location Results			
	z (m)	r (m)	SINR (dB)	PBR (dB)
Bartlett	76.1	5300	10.70	5.41
MVDR	74.8	5300	11.57	6.49
MFP-CPC	74.8	5350	14.32	9.16
